# Collectanea Medica, Consisting of Anecdotes, Facts, Extracts, Illustrations, Queries, Suggestions, &c.

**Published:** 1816-01

**Authors:** 


### COLLECTANEA MEDICA,

CONSISTING OF

ANECDOTES, FACTS, EXTRACTS, ILLUSTRATIONS, QUERIES, SUGGESTIONS, &c.

RELATING TO THE

*History or the Art of Medicine, and the Auxiliary Sciences.*

---

### *Memoir on the State and Composition of the Atmosphere, considered as the Cause of the Hospital Gangrene, or Putrescence among the Wounded.* By Professor S. J. Brugmans, of Leyden.

*(Concluded from our last.)*

### Chapter IV.—*Of the Means capable of preventing that State of the Atmosphere which immediately gives rise to Hospital Gangrene; and of correcting it as speedily and as efficaciously as possible, if it is already developed.*

SECTION 56. This chapter naturally divides itself into two sections: the first on the prophylactic means; the second on those by which we may oppose contagion when it exists.

*Of Prophylactic Means in general.*—§ 57. After what has been said respecting the occasional causes which may prepare, favour, and produce, the development of the miasma, it will not be difficult to point out the most proper preservatives. I shall endeavour to detail them succinctly, and to show their value by actual observations.

The contagious matter is engendered in the air in which the patients exist, or, with greater certainty, is brought from without. See Chap. II.

*Best Method of preventing the Development of the Miasma.*—§ 58. The contagious matter proceeds from the exhalation of the human body, particularly when attacked with disease, and placed in an atmosphere not often renewed. The development is also favoured by the whole series of causes enumerated in § 28.

The methods by which we may prevent the removal of patients into hospitals from occasioning gangrene, or dissipate the causes of it when they have taken place, depend so much on the management of the hospital, and the mode of general treatment, that, precisely and completely to explain every thing to be done, in order to banish this dangerous disease, it would be necessary to enter upon an infinity of details; but we shall suppose that the proper management of hospitals is well known, and that it is only necessary here to dwell on the principal points. These are as follow:

§ 59. The hospital ought to be situated in a dry place, removed as far as possible from marshy ground, stagnant waters, and every collection of putrefactive matter, and, consequently, in a wholesome atmosphere.

If the wards are according to the dimensions already given, every patient will have, as he requires, at least 500 cubic feet of air. The renewal of this air is not less indispensable. Ventilators ought to be open as much as possible over the beds, and placed opposite to each other: the breadth of the whole taken together ought to be nearly equal to the half of the length of the ward. Besides these, it is absolutely necessary that on a level with the floor there should be air-holes, about a foot square, and which may be shut at pleasure by sliding doors. These air-holes ought to be at the distance of 10 or 14 feet from each other.

For wounded patients, rooms with a southern and eastern exposure are preferable.

Finally, the beds should be detached at intervals, as we have fixed in § 32; and the bottom of every bed should be elevated 14 or 16 inches above the floor.

§ 60. By attending to these particulars, and when the surgeons and assistants see that the air-holes and windows are open at the proper time, and not shut all at once on any account, even in winter nights, I dare venture to say that the air of the hospital will never be infected so as to occasion the slightest apprehension of this dreadful disease.

§ 61. The lighter strata of corrupted air will go off by the ventilators, and that which is heavier than pure atmospheric air, and is by far the most noxious, will go off by the air-holes. If a portion of this heavy air remains in the ward, it forms a stratum on the floor much below the patients' beds. In the case of hospital gangrene, this heavy air is infinitely prejudicial; thus, all things being equal, this disease is much oftener observed in low beds. It is never so frequent as when the patients are laid on mattresses or palliasses on the floor, which is too often the case in time of war.

§ 62. Two instances will here suffice to confirm the utility of this arrangement, considered as a preservative process.

1. When in wards of considerable size, the windows, air-holes, and beds were placed as described, and, finally, when the nurses did not neglect to renew the air, I have been uniformly convinced, by an infinity of experiments, that the air inspired by the patients was neither less fresh, nor of any other composition than the common air, whatever might be the season of the year, the hour of the day, or circumstances of the patients.

2. With such precautions, I have not witnessed, during the long space of eighteen years, during which I have been at the head of the army medical department, the appearance of the hospital gangrene.

Among a great number of facts which I might detail, the following will suffice.

In the year 1799, we received into the military hospital of Leyden 4000 wounded men. In this number there were many whose wounds secreted an enormous quantity of pus. We had, at the same time, thirty-four with fractures of the femur, complicated with gun-shot wounds, and all were placed in the same ward; besides which, there appeared several diseases of a bad character.

The situation of the hospital was far from good: we did not, however, perceive the slightest train of hospital gangrene. The renewal of the air was the chief cause of this success; and the indefatigable vigilance of a meritorious individual received the grandest recompense to a noble mind, that of having preserved the lives of hundreds.*

Military surgery requires no more energetic method of preserving the sick and wounded, than that of properly airing the wards. Every other disinfection or artificial amelioration of the air is superfluous, and even noxious.

§ 63. To the above grand preservative means of keeping the wards always filled with pure atmospheric air, we may add the following:

Remove every thing which tends to putrefaction and gives a bad

* Dr. Stark, then Surgeon-major in the Military Hospital of Leyden, and afterwards Member of the Dutch Army Medical Board.

smell; preserve, as much as possible, from all filth, the rooms, bedsteads, bedding, clothes of the patients, their bodies, &c.

A mattress which has been used for whole years without being cleansed, becomes like a sponge imbued with animal emanations, and it can scarcely be imagined to what a degree such a mattress will corrupt the air in a few hours.

§ 64. Let the food be sufficient to give strength, or at least to preserve it. Too great economy will soon kill a wounded patient.

Tonic drinks are of the first importance: the water ought to be always perfectly pure.

The surgical cases ought to be regularly dressed, that the pus may not remain too long on the ulcers, or putrefy in the bandages. In all cases we must remove from the rest of the patients those from whose wounds the pus is fetid, of a bad character, or too abundant. Neglect of this precaution has frequently spread hospital gangrene through a whole hospital.

§ 65. We may also reckon among the preservative remedies the continual vigilance of the surgeon to remove from the vicinity of the wounded all feverish patients, and particularly those who are seized with diseases which lessen the vital powers, such as malignant fevers, next, eruptive diseases, fluxes, &c. We ought also to separate from the rest of the wounded all those who are attacked with any gangrenous or malignant ulcers, of whatever description.

§ 66. Finally, among the causes which favour the hospital gangrene, I have still to include those passions of the mind which depress, and even destroy, the vital powers. If it is not in the power of the surgeon to dry up the source of these moral affections, he may always, by his humanity, mildness, and the interest which he takes in the fate of his patients, contribute greatly to the recovery of many who daily sink under wounds of little importance in themselves, solely on account of some galling mental affliction.

*Means of opposing the Propagation of the Disease.*—§ 67. We have seen that this disease is communicated through the medium of the atmosphere, and in a particular manner, by the application of infected pus to a healthy ulcer.

When a patient is attacked with an ulcer in which we discover the slightest symptom of hospital gangrene, or even any precursory sign of this disease, it is important above all things to remove him from the rest, to take care that the corrupted air may not penetrate into a healthy ward, that no patient from the clean wards shall visit those who are attacked with hospital gangrene, and that the nurses shall not heedlessly run out of one ward into another.

The lint which has been used for the dressings, and which is saturated with pus, ought to be buried under ground, or thrown into the fire. It is also to be wished that the linen of which a bandage consists, when it is stained with pus, should be destroyed, since the ordinary washing, and even long bleaching, are insufficient to completely remove the contagious matter.

It is of the last importance that the mattresses, bed-clothes, &c. of of a patient affected with hospital gangrene should not be given to another before undergoing a necessary purification.

Finally, the surgeons ought to take care never to put their fingers or instruments on a well-conditioned ulcer, if they have been touching a malignant ulcer, for, whatever M. Richerand may have seen in this respect, the importance of these precautions is clearly demonstrated by the experience of many other enlightened observers, by facts of which I have been many times an eye-witness, and even by the nature of the disease itself.

*Methods of correcting or destroying the Miasma.*—§ 68. After having treated hitherto of the means necessary for preventing the development and spreading of the miasma, we shall now suppose that the hospital gangrene exists, and that, consequently, the ulcers give out the miasma, which, as already observed, is communicated to the atmosphere. The question is confined to the inquiry, what are the physical and chemical methods which we may oppose to the disease? I, therefore, omit the medico-chirurgical treatment proper to be employed.

§ 69. The instant the malignant nature of the ulcers is perceived, we must examine attentively into the causes which may have occasioned it. In general, one or more of the above-mentioned causes will be discovered, and in this case they must be removed as speedily as possible. The origin of the contagion will rarely escape the detection of an experienced practitioner.

§ 70. The methods now to be resorted to are of two kinds. They tend to remove the infected atmosphere with all that is engendered in it, or rather to annihilate the miasma which already exists.

§ 71. In order to remove the miasma when once developed, it is not sufficient to open the doors, windows, &c. in order to admit fresh air: we must have an entirely new atmosphere. Trials frequently repeated, and always attended with the greatest success, leave me no doubts as to the efficacy of the following process.

We must begin by furnishing the patients with proper clothing, which must be changed from time to time; they are then to be laid on double palliasses, recently made, and not upon sacks filled with wool or hair; at the same time that they are covered with clean bed-clothes. Afterwards, if the season will admit, the beds are to be carried into the open air, and arranged, according to the direction of the wind and other circumstances, in the vicinity of a hedge, a wall, or any other inclosure. At the first view, many people will be afraid to place feeble patients thus in the open air; but let them be perfectly easy as to the event. What practitioner has not remarked the surprising efficacy of fresh air in the most virulent stage of small-pox, as well as in the most dreadful convulsions? The fresh air acts not less miraculously on patients attacked with hospital gangrene. If the rain or wind prevents entire exposure, tents ought to be erected, or an open gallery procured, cured, in which to place the beds. Let not expences or difficulties deter us! What are expences and difficulties in comparison with a single human life? our business is the salvation of thousands.

Let the patients remain in the open air the whole day, until evening comes in: during this period the ward from which they came must be purified in the manner I am going to describe, or rather a ward not infected ought to be selected for lodging the patients during the night; next day they should be carried into the open air again.

§ 72. I have frequently derived the most inestimable benefits from this process. It will be sufficient to mention, that in the summer of 1807 the hospital gangrene broke out with all its fury at Leyden; and, in a single ward, upwards of sixty individuals were attacked with it at once. These patients underwent the above treatment, under the active vigilance of M. Luhrman, a most valuable and experienced practitioner at Leyden. The success exceeded our most sanguine hopes.

If the situation of the hospital, the season of the year, or other weighty considerations, absolutely forbid the patients being carried into the open air, the wards must then be changed. A second ward ought to be properly aired, for carrying the patients into it early in the morning; at the same time the first must be purified, as we are about to explain; next day the patients are to be placed in their former ward, and the new one is to be purified in its turn for their reception on the third day. In this manner the wards are to be changed, until not a vestige of contagion remains.

§ 73. The second process, which I have now to describe, has for its object the extinction of the miasmata.

Make a very large fire, scour the wards with a great quantity of water, whitewash the walls with lime, not to mention opening doors, windows, and air-holes; for all these methods, however justly relied on for purifying the air under any other circumstances, would not be sufficient to carry off, or rather to destroy, the contagious matter.

Guyton has particularly demonstrated that corrupted air cannot be corrected by fumigation of aromatic substances, such as oils, resins, fruits, or oleaginous seeds; nor by the detonation of gunpowder; nor by the evaporation of vinegar: it is, therefore, needless for me to recur to this topic.

§ 74. The only method of attaining the proposed object is, in the first place, the vapour of nitric acid, but, above all, that of the oxygenated muriatic acid gas. Guyten, in discovering the property of this last gas in destroying all kinds of noxious animal emanations, and, consequently, annihilating every contagious animal substance, has deserved well of mankind.

§ 75. It would be superfluous to treat here on the utility of these fumigations: they are known to the whole world by the writings of Guyton, Pinel, Smith, Odier, Rollo, Des Genettes, Paroletti, Cruikshanks, Poggi, &c.

Their united observations lead to the conclusion that these fumigations destroy, in a particular manner, the miasma of small-pox, of the venereal disease, protic diseases, the hydrophobia, yellow fever, and, according to the observations made by Fleury in the hospital of Cherbourg, they also destroy the miasma of hospital gangrene. (Ann. de Chimie, t. 46, p. 118.)

§ 76. In order to fulfil my promise, I shall confirm, by some experiments, the efficacy of the Guytonian fumigations in extinguishing the contagious matter of hospital gangrene; and I shall afterwards give the method which appeared to me the most convenient of applying this process to practice.

§ 77. I placed in the hydropneumatic apparatus a very large glass, filled with an air strongly impregnated with the emanations from a pus which was secreted from ulcers infected with hospital gangrene. A small number of the bubbles of Guytonian gas were sufficient to take all smell from the air, after being some hours in contact with the water.

I impregnated distilled water with exhalations from the above pus, according to § 39. By this process, the water acquired the peculiar smell which we have so often mentioned. As soon as I had mixed the above-mentioned gas with it, the water lost the smell of the pus, and, when kept for a long time, it did not go into putrefaction.

Although corrupted air may engender hospital gangrene, and although the latter greatly favours putrefaction, nevertheless the Guytonian fumigations deprive it totally of that tendency to putrefaction, as may be seen from the experiments mentioned in § 47.

I washed some lint, imbibed with pus, slightly in clean water: after having expressed the water as much as possible, I exposed it to the fumigation; afterwards I washed it, dried it, and aired it again, until not the slightest smell of muriatic acid gas remained. I used the lint on well-conditioned wounds without inducing hospital gangrene.

Finally, the power of this gas was still further demonstrated by the great use it was of to me in carrying off the above-mentioned miasma, as well as other contagious matters. I used it in the following manner:

§ 78. In order to disinfect clothes, coverlids, sheets, palliasses, mattresses of woollen or hair, the following apparatus is necessary.

Near every hospital let a small room be built, for instance, 10, 12, or 16 feet square, entirely devoted to fumigations, and for this purpose hermetically closed on all sides. Two or three feet from the floor, place a grating of thin wooden lathes, separated two inches from each other, and covering the whole surface of the room. At a height of four, five, or six feet above the grating, let ropes or stronger lathes be fixed, in order to fasten the heavier pieces of cloth which are to be purified. On the grating itself, wool, hair, and other objects of small size, are to be placed. In the the lower part of the door, make a small aperture, 14 or 16 inches square, in order to introduce on a plank, into the middle of the room, the apparatus necessary for extricating the gas.

This apparatus consists of a chafing-dish or brasier, on which is placed turf, well lighted, and covered with ashes; over this place a glazed earthen pot, containing the above mixture of muriate of soda and oxide of manganese, dried, and afterwards diluted in a little water; then add the necessary quantity of sulphuric acid, and stir up the whole quickly. By means of the plank, the earthen pot is to be introduced under the grating, where it is to be left for two or three hours, taking care to close accurately the door and passage hole. The mixture may be withdrawn from time to time, and stirred, if the vapours are not very abundant; or more of the ingredients may be added; or the fire rendered more active, as the case requires. In this way the gas fills the whole chamber, and penetrates thoroughly every article which it contains. The whole may be kept shut up for twenty-four hours, afterwards giving free admission to the air. The fumigated subjects may be removed, which, after having been deprived of their pungent smell, by washing, or simple exposure to the air, may be regarded as free from all miasma:

With respect to the purifying of mattresses, they must be taken to pieces, and their contents exposed separately to fumigation.

§ 79. It now remains for me to show the method of disinfecting the wards themselves by this fumigation.

It is always best to remove the patients. When the walls are afterwards whitewashed, and the boards scoured with water in the usual manner, the fumigation may commence.

For this purpose, let the apartment be accurately closed, and in the centre, ten feet apart, let brasiers be placed, containing the fuming mixture already described. The whole ward will be instantly filled with vapours, resembling a thick fog. The whole may then be allowed to remain for twenty-four or forty-eight hours, after which the doors and windows are to be opened, the walls and glass windows well washed, and the flooring also; and for a few days free access must be given to the atmospheric air, when we may be completely satisfied that no more miasma exists in the ward.

§ 80. If it is impossible to empty the ward, a less perfect fumigation may be resorted to: place in glasses or small pots, capable of containing from eight to ten fluid ounces, a small quantity of the fumigating mixture, and heat it so slowly that a very slight vapour may be diffused through the ward. Continue thus to diffuse as much vapour as the patients can support without coughing too much; and, at the same time, give a free admission to the atmospheric air. In this way the Guytonian fumigations may be very salutary, particularly if other disinfecting processes be conjoined with them. This partial fumigation has frequently been of great service in preventing the miasma of a bad ulcer from being communicated to other individuals, through the medium of the atmosphere.

atmosphere. Nevertheless, if the contagion reigns throughout a whole ward or building, this slight and partial fumigation will not be sufficient, nor any thing less than the measures above mentioned.

§ 81. On a review of the whole, it will be seen that the success of every method suggested depends on the person who has the charge of the hospital. It is also evident that *a mere surgeon* is not the person required: it is necessary that he should attend to both physic and surgery, regarding them as two inseparable branches of the same science. The first period of the disease in question, and which it is so easy to stifle by the simplest efforts, will escape the observation of a person who has not a profound knowledge both of medicine and surgery. We may add, that even in the cure of the disease, internal remedies are frequently as efficacious as external applications.

The director of the hospital ought also to be well versed in physics and in chemistry; otherwise how can he know the connection of the phenomena which explain the state of the atmosphere, observe the characteristic signs of its purity or infection, and investigate the causes on just principles?

Finally, the practitioner in question ought to be endowed with a more than ordinary vigilance; his attention should embrace every thing connected with the hospital; let his indefatigable zeal never rest night and day, and, not content with performing well his own duties, let him also watch over his subalterns, and especially the nurses.

In the hands of such a man, I venture to believe that the methods prescribed will not only diminish the danger of hospital gangrene, but banish it altogether. Too often it must be regretted that the events of war do not permit. They are often such as are inconsistent with all the rules of art. But let us be silent. This is no part of our plan; and happy shall I feel if I have succeeded in throwing some light on the nature and causes of this formidable disease, and on the best means of removing it.

"*Naturalem causam quærimus et assiduam non raram et fortuitam.*"

---

The Report of the Madhouses has, at length, been so hacknied, and with so little delicacy, that we shall rather select those passages which have not hitherto, as far as we know, been submitted to the public, than attempt to surprise our readers by disgusting histories of cruelty, inattention, and ignorance.

"*Powers of Commissioners, &c.*

*Jovis,* 11° *die Maii,* 1815.

The Right Hon. George Rose *in the Chair.*

Dr. Richard Powell *called in, and examined.*

You are a Fellow of the College of Physicians?—I am.

And Secretary to the Commissioners appointed under the Act of the 14th of the King, for the regulation of Madhouses?—Yes; and have been so from September 1808.

Do

Do the College of Physicians annually elect five Fellows for visiting Madhouses within seven miles of the metropolis?—They do; according to the directions of the Act, and at their general meeting in September.

Do the Commissioners meet as prescribed under that Act, to grant licenses?—They do, according to the directions of that Act.

Do the Commissioners visit the houses once a year, and occasionally at other times?—They do; all houses once, and, where there appears a necessity, more than once.

Do they, on such visits, examine minutely into the state of the houses, and into the condition of all the apartments in them, and as to the care of the patients?—Yes.

Do they enquire into the medical treatment of the patients?—Not particularly.

Do they at all enquire into the medical treatment?—Into the medical attendance, certainly; that is to say, it is very frequently asked, Who attends this patient? but, into the propriety of the medicine administered, or into the medical treatment of the patient, they do not go; nor do they make this enquiry in every case.

Do they make minutes of their observations in those examinations?—They do; their present minute-book, which I have been ordered to bring with me, I beg leave to lay before the Committee.

[*Dr. Powell produced the same.*]

The Commissioners are sworn of course?—They are; every part of the Act, I believe, is fully complied with; it is the wish of the Commissioners that it should.

The minutes contain every particular which the Commissioners think deserve their notice, with their observations thereupon?—After the visitation, all the circumstances respecting every house visited on that day, are read over; and they determine upon the general minute that shall be entered upon the minute book, from the loose minutes taken at the time, and from their own recollection.

Have circumstances occurred tending to impeach the character of any of those houses, upon the visitations made?—Certainly. Every fault that is found, impeaches the character; and there are many noticed in that book.

If any discovery is made deserving censure or animadversion, is that stated in a paper, and hung up in the censor's room of the College?—Very seldom; because the method of publication by such mode of hanging up has been thought to be completely inefficient: It is thus hung up in a private room, into which few persons go; but, if any minute is made containing censure, or finding fault with particular conduct, the secretary is usually directed to write to the keeper, and to inform him of the circumstance: And if the house has been ill conducted, the Commissioners make a point of visiting again very speedily, in order to see whether the same objection still exists.

Have instances occurred of keepers refusing admittance to the visitors?

visitors?—No, I believe not; not within my knowledge, certainly.

Is an exact account kept of the whole of the proceedings of the Commissioners?—The minute they agree upon is entered; it is again read to them at their next meeting, and they sign it after consideration, as will appear from the book before the Committee; of course very many things occur during a visitation which are not mentioned in the minutes.

Have you reason to believe that regular accounts are transmitted of patients in the houses within seven miles of London, within three days after their admission?—There are instances to the contrary; but in general I believe they are very correctly returned.

When instances to the contrary have occurred, what has been done upon it?—If the omission appeared to have originated merely from error, the Commissioners have reprehended the keeper, and directed greater attention for the future; but in glaring instances they have ordered prosecutions.

In what instances can such omission have originated in error?—The confusion, for instance, between parish paupers and patients that are paid for, parish paupers not being returnable under the Act.

Have not the keepers of all those houses full information of the nature of their duty, which distinguishes between the parish paupers and the others?—I suppose they have, as far as the Act can give it.

Then how can it have arisen from error?—The Act is obscure: I can point out instances in the register where the return has been delayed to four or five days, instead of being made within three.

Do you mean to say those are the only cases where they have not complied with the Act?—Certainly not; there have been cases in which the Commissioners have even directed prosecutions.

Have the Commissioners directed prosecutions in all cases, except those of a trivial departure from the law?—No; this has depended upon their investigation of the facts, and I can probably answer the question better by a fact. A person was found confined in a house: one of the Commissioners had seen this person, who was admitted into this house without any certificate; the Commissioners visited, and found that he was so; he had been there but a little time, and his conversation then was exceedingly correct; they found that a statement had been made to the keeper, that the Commissioner had so seen him, and believed him to be insane; he took this report for a certificate, which it was not; but this was merely a mistake, without any intention of concealment, and so far excusable. This man, in consequence of the reprehension of the circumstance by the Commissioners, got at large, but he was insane, and very soon in confinement again; his was insanity of conduct, not of conversation.

Are you aware of lunatics being admitted without a certificate at all, in any case?—No, I am not: when I say that, I beg to state that I could refer to an instance where a lunatic has been received and confined without any certificate, or without any return, turn, and the Commissioners have ordered a prosecution against the party; but, as a general conduct, certainly not.

As far as you are enabled to speak upon the subject, have those orders for admission been properly attested?—Yes, generally so, certainly; I would beg here to state to the Committee, that in consequence of the irregularity of such orders, the Commissioners directed papers to be printed and used, containing the medical certificate which ought to be required, and which they expected to find; and also blank returns of the circumstances required by the Act to be filled up by the keepers; and which are now mostly used, but not always, as they have no power to compel it.

[*The Papers were delivered in, and read.*]

'Medical Certificate.—For the reception of a patient into a licensed Lunatic house.

In consequence of sufficient personal examination of I hereby certify to be of insane mind, and I am of opinion that suitable confinement of in a house licensed for the reception of Lunatics, is necessary and proper.

Signed and sealed by

Dated this day of

To Mr.

N.B.—To be signed and sealed by some Physician, Surgeon, or Apothecary, and his residence and branch of the profession to be added.'

'Name of the person received

Date of admission into a Lunatic house

Name and residence of the friends or other persons by whose direction the lunatic is received

Name and residence of the physician, surgeon, or apothecary, by whose order, signed and sealed, such direction is given

Signed

To the Secretary for licensing houses for the reception of lunatics.

To be left with the Beadle of the College of Physicians, Warwick-lane, London.'

Do you always compare the number of persons in confinement in any given house, with the number of certificates?—No; it is impossible to do that.

Why is it impossible?—We have a file brought with the certificates jumbled together upon that file; patients have gone out of the house, and the certificates remain upon the file; in short, we only take some of these papers and collate them, and see that they accord with our registers and returns; we do not attempt to go through the whole, but take one indiscriminately, and see that that is correct, and from finding three or four so, we rather infer that the whole are so.

Do

Do not you think the mode of entry might be so arranged and ticketed, on filing those certificates, that it might be easy to compare the certificates with the persons then under confinement?—Yes; but we have no power to direct it.

Of course, that would prevent altogether almost the possibility of a person being there, for whom there was not a proper certificate?—We are so far secure of the certificate, that the name of the medical person signing the certificate, as well as that of the friend of the patient, is entered in our book.

You are not sure that the man may be there?—No, the patient may be gone; we know nothing of him in the register beyond his admission; the Act provides for nothing more.

Recognizances are taken always before the licenses are granted?—They are in the Court of Chancery.

Have the Commissioners regular returns from the country, with respect to the number of houses, and the names of the patients confined in them?—They are imperfectly made in very many instances: many houses in the country do receive patients and never return them at all; we communicate this to the Clerk of the Peace, when we know it; he uses his judgment, and there the matter ends. The Commissioners receive no return of houses as they are licensed; the only knowledge they have of a house even being in existence in the country, is the receipt of a return, if made by the keeper, of the admission of a patient therein.

Have many returns been made to the Quarter Sessions, so far as you know, of minutes of the state of houses for the reception of Lunatics, at the visitation of licensed houses within the respective counties?—The Act directs that the visitors in counties *may*, if they think necessary, make minutes in writing. In case such minutes are made, then they are directed to return a copy of the minutes so made to the secretary; but they may visit without making any minutes at all. The copies of minutes which have been so returned, are certainly not from all those counties containing houses.

In the event of a license being withdrawn from a person keeping a house for the reception of insane persons, on account of gross misconduct, have the Commissioners power to refuse that man a new license, if he should apply for one the next day?—Certainly not, under the Act. I know no instance, however, of a license ever having been taken away.

In the various visitations the Commissioners have made, has no instance occurred, which, in the opinion of the Commissioners, would have justified the withdrawment of the license?—I cannot immediately lay my hand upon a minute of the sort; but I remember one, where the Commissioners would have thought it justifiable to have refused the license, if they had the power: they never have withdrawn it.

Has no instance occurred within your observation, of circumstances falling under the cognizance of the Commissioners, which would have justified their withdrawing the license?—Probably many circumstances of ill conduct would have done that, and there have been abundant instances of such, but the Commissioners have never in fact withdrawn the license; how far they would have considered themselves justified in doing it, I cannot say.

Do you conceive that the provisions of the Act of the 14th of His present Majesty, are sufficient for carrying into effect the purposes intended by that Act?—I do not; the visitations have done an immensity of good; but the Commissioners have not sufficient powers.

State to the Committee the deficiencies, so far as they have fallen under your own observation?—May I be permitted rather to refer to the copy of a letter to a Peer, which will point out the chief deficiencies of the present law.

[*The Letter, together with one to the Chairman of this Committee, were read, as follow:*]

"My Lord,

The Commissioners under the Act for regulating Madhouses in London, and within seven miles of the same, and within the county of Middlesex, beg leave to offer to your lordship their acknowledgments for the communication made to them through Dr. Pemberton, relative to the above Act. The suggestions and alterations proposed by your lordship, meet their entire concurrence; at the same time, as they relate only to local circumstances, and as the Commissioners are aware of many other defects, more immediately falling under their own cognizance, they have taken the liberty of suggesting them, in order that, when embodied with the alterations proposed by your lordship, a complete revision of the Act may take place.

The Commissioners beg to state, that the subject has heretofore, on various occasions, been taken into their most serious consideration; that the alterations they will have the honour of submitting, have been maturely weighed, and severally laid before counsel, for their opinion; that the whole have been digested into form; and of the opportunity now afforded by your lordship's offer, they feel it to be important to the interests of the public that they should hasten to avail themselves.

The Commissioners, therefore, propose to submit to your lordship, in the first instance, a very general view of the sufficiencies of the Act in its present form; of the modifications of which it is susceptible; and of the new matter which it may be thought proper to introduce therein. They deem it unnecessary to detain your lordship with a detail of inaccuracies of verbal expressions in the Act, although such are numerous, and productive of much inconvenience in the execution of it, because any such alterations can be better explained, and more easily made, if the subject shall hereafter receive minute consideration. For the same reason, the Commissioners will also omit some charges which refer to convenience alone, such as the manner in which the oaths are directed to be administered (p. 1093) and the place of their meeting, which is limited to the Hall, or some other convenient place in the College of Physicians (p. 1094).

That various defects in the provisions of the Act generally do exist, appears from the Minutes of the Commissioners; the necessity for their frequent recourse to legal advice, and the publications of the proceedings of Courts of Law, and your lordship's recent information in the county of Wilts, is one proof among many that similar defects are felt in the more distant counties of England. That the verbal expression of those provisions is not in all instances clearly made, may be illustrated by an opinion of the late Lord Kenyon in 1782, which begins thus, 'I cannot give a receipt to provide for the inaccuracies of an ill-penned law.'

The first point to be considered in order, is the granting of licenses, which (page 1095) the Commissioners are required to grant to all persons who shall desire the same; and, by the opinion of counsel, they have been advised, that no impropriety of conduct, not even a previous conviction of offences under the Act, or the forfeiture of the license under one of its clauses, will, as the law at present stands, justify their refusals.

The Commissioners (p. 1095) are also restricted in granting licenses to one day in the year: it would be advisable that they should possess the power to grant them at any other time, provided that the licenses all terminated on the usual day of the year that the other licenses are granted for. It is to be observed, that the Magistrates of counties have the power of granting licenses at any General Quarter Sessions.

The sums to be paid by persons taking out licenses are now regulated by the number of houses, and by one single division only (as they may be *ten* in number, or *more than ten* (p. 1095) of the number of patients who shall be confined in each house. Under the present regulations, therefore, provided they be contained in one house, twelve patients or eighty patients require the same license.

The Commissioners imagine, that a scale may be formed according to the number of patients contained in one establishment, rather than the number of *houses* of which it may consist, which may not be more burthensome to the keepers in general, not much more productive to the fund from which the expenses of the commission are defrayed, and yet be more equal and just to all parties than the present arrangement. Such an adoption, too, will obviate some objections which occur in the execution of the Act, and do away the motives which exist to confine a great number of these unfortunate persons in a small space. Further, as it will hereafter be proposed that parish paupers shall not continue to be, as they are at present, deprived of any of the benefits of the Act; and as such lunatics are received at lower prices by the keepers, it might be proposed that houses licensed for their express reception, should pay for their licenses *only half the sum* determined upon for others.

As licenses are now granted (p. 1095), any person may require one; and it is true that he is rendered by his recognizance amenable for the conduct of the house; but such licensed person may not reside in the house; he may, if he pleases, entrust the charge thereof to a servant; and further, although one person only may take out the licence, it frequently happens that others also have a trading interest in the concern; it seems, therefore, proper and reasonable that these circumstances should be regulated, that all the parties interested should be answerable for the conduct of a Lunatic house, and join in the licence; and also that *one of the parties* to whom any house is so licensed, should reside upon the spot. Perhaps this provision might be enforced by a penalty attached to disobedience of it.

Again, the present Act allows the confinement of one Lunatic in a house without any license (p. 1092); and this provision looks very properly to the accidental occurrence of Insanity in private families, and to their security, under such afflicting circumstances, from vexation or interruption; but this privilege is construed to extend much further, for persons consider themselves to be justified by it in confining any number of patients in distinct houses, and even in contiguous ones.

The superintendance of the Commissioners is thus evaded, and they recommend that provisions be introduced into the Act, by which the practice may be prevented. As also the words of the Act at present stand (p. 1100), the penalty will be found to be incurred by those who shall 'admit, harbour, entertain, or confine, any (person as a) lunatic,' which circumstances of actual occurrence render it expedient to change into 'admit, harbour, entertain, or confine any lunatic,' omitting the words in a parenthesis.

The line of conduct to be observed by the keeper of the licensed house, should seem to follow next in order; and the directions of the Act, by which these are to be regulated (p. 1099), will require very material alterations.

Pauper Lunatics, sent by parish officers, are exempted from any returns on their admission into a Lunatic house, such as are required for other patients; and the Commissioners have found also, that, as the law at present is interpreted, no medical certificate is required to justify their confinement; this practice they have endeavoured, but not effectually, to do away; and the fiat of the parish officer alone is thus sufficient to consign a person to confinement.

The Commissioners conceive it will be thought right, upon every principle of humanity and justice, that all exemptions of this kind should be done away, and more particularly so, because it has been sometimes contended, that even private patients, if they be but admitted upon the same low terms as parish paupers, may be classed with them, and exempted from returns, and the other provisions of the Act.

A certificate of some physician, surgeon, or apothecary, as to the insanity of the patient, is necessary (p. 1100) to justify the admission of such patient into a licensed house, to which description it ought, in the present state of the profession of medicine, to be added, some physician, surgeon, or apothecary, *legally qualified to practise as such.* The nature of the certificate should also be definitely expressed, and affirm, that the medical person who signs it has *actually visited* the patient. The London Commissioners have recommended the form which is attached to this Report, and on their visitations examine and compare the files of such certificates, and they would advise that a similar form should be added in an appendix to the Act.

The keeper is also bound (p, 1100) to return the name or names, and place or places of abode of the person or persons by whose direction the lunatic is admitted; and as the certificate of the medical person is directed to be signed and *sealed* by *him*, so ought also the direction of the friends of the patient, which is not at present required by the Act.

The returns to be made by the keeper to the secretary in London, on the admission of any lunatic, are, by the present Act, directed to comprise certain particulars, but these were found to be very defective, until the adoption of the form annexed to this Report; and there is still considerable difficulty in persuading the keepers of some houses in the country to employ it; It is recommended, that a similar form be adopted in the Appendix to the Act. As the return now stands, it will be seen that, besides the patient, there are three distinct parties concerned; i. e. the friend or friends who desire, the medical man who advises the admission of the patient, and the keeper or person to whom the house is licensed; and it is particularly advised that these three characters should be kept separate, and that a clause in the Act should prohibit every individual from signing the return in more than one capacity; and that no keeper or other person interested in a licensed house should sign a certificate for admission into it, in the character of a medical practitioner.

Further than this it seems necessary, that the observance of this most important point of medical certificates should be enforced by a penalty, which ought to be imposed upon the parties concerned, if any such medical certificate shall be proved to be improperly granted, and without personal examination of the patient: And here the Commissioners cannot but approve your lordship's suggestion, that a *plan* of the whole house to be licensed, and its respective rooms, should be delivered in at the time of first taking out a licence, and as often as any change shall take place, or the Commissioners require the same; it will afford the Commissioners an information which is important, and which they do not at present possess, further than by the knowledge they acquire by repeated visitations; perhaps the accuracy of such plan will be better enforced by a penalty for every false return, than by an oath on the part of the keeper.

It

It will also be proper to direct the keeper to make still further returns of the state of their houses, besides the returns, within a certain number of days, of patients received therein; *and also at the time of taking out the annual licence, that the keeper should deliver to the secretary an account of all the patients then entertained under his care*, and that he should make a return to the secretary, within the same number of days, *of discharges, or deaths, or other changes*, which may occur therein; and these circumstances, with their several dates, ought to be added, in separate columns, to the corresponding names in the register, which at present can only account for the admission of patients, and affords no means of obtaining that further information, which is so often sought for as important.

The visitations to be made by the Commissioners, and their several duties and mode of election, come next (p. 1099), and will probably require no alteration; but it is suggested, that, if the mode of paying for licences according to the number of patients, as before stated, be adopted, it will also be proper that the remuneration to the Commissioners should follow the same rule, rather than, as at present, one guinea should be paid to each for every house visited; there should also be a clause to empower them in their visitations to liberate from confinement any person whom they shall have sufficient reason to consider as of sane mind, or improperly confined, which at present they do not possess, as under many cases of such occurrence, the slower though regular process of the law must, from the time it requires, be a source of infinite distress to individuals in such a situation.

The Commissioners in London are by the Act (p. 1092) elected annually on the last day of September, the former Commissioners then quit their office; the new Commissioners meet for the purpose of granting licences on or within ten days of the third Wednesday in October, which licences are to commence on the 20th of November following. Now either the licences should commence on the day upon which they are granted, *or the new Commissioners should be specially empowered, if they please, to visit during the remaining term of the licences granted by their predecessors*, and not to leave, as seems at present to be the case, seven weeks, i. e. from September 30th to November 20th, without any regular superintending power.

It is enacted (p. 1097) that any part of the Report of the Commissioners which conveys censure or animadversion upon any house, shall be *hung up in the Censor's room of the College*, to be perused and inspected by any person who shall apply for that purpose; and the insufficiency of this provision to any good purpose must be manifest. It is therefore proposed to alter it, and to direct that a copy of such part of the Report as conveys censure *shall be communicated to the keeper of the house, and also entered in a separate register, to be kept in a convenient place at the College, for public inspection.*

The

The Commissioners ought also to have a power vested in them by the Act, of taking recognizances at the time of granting licences; and the clause giving them this power should also require, that they be returned to the Quarter Sessions for the county, a power which the Commissioners for other counties at present possess.

The application of the Act to the counties of England and Wales generally, might demand various considerations according to local circumstances; but it is supposed that any such will not require much deviation from those which the London Commissioners recommend, founded upon ample experience.

In relation, however, to the houses in counties, it may be observed, that (p. 1102) the Keepers are directed to make a return of the lunatics they admit, to the secretary to the London Commissioners, within fourteen days from such admission, which names, &c. are to be entered in a separate register. Now, as the Act stands, such entries must be imperfect and insufficient; these returns from the keepers are the only intimations of the existence of such houses which the secretary receives, and even these are, in some instances, wholly omitted, and houses exist, and no returns are ever received from them. It would therefore supply a material check upon such irregularities, if the *Clerk of the Peace* for the county or place where such houses are licensed, shall be obliged, under a penalty, to *return a list to the secretary in London, of such licenses, within a given time after they are granted.* And it is further presumed, that the purposes of the Act would more effectually be promoted, if the Commissioners for counties were directed to make regular *minutes of the state and condition of houses, and of the number of patients confined therein, and to transmit copies thereof to the secretary in London,* under certain penalties, rather than that such minutes and transmissions should be left so much at large, as is done by the terms of the present Act.

There is furthermore another general provision of the present Act (p. 1104), which seems to require some modification. It is there provided, that the Act shall not extend to any of the *public hospitals* within this kingdom. And the Commissioners suggest, that this should be confined to *pauper* lunatics admitted into such institutions as objects of charity, and not extended to those who pay, and sometimes largely, for their accommodation and treatment, as is at present the case in a variety of places, which shelter themselves under this designation, and receive patients of all descriptions who may offer.

The Commissioners beg leave finally to observe to your Lordship, that any illustration, by cases of actual occurrence, of the opinions contained in this general statement, is, for obvious reasons, passed over; but, if it should be deemed necessary, there is no point here mentioned which may not be supported by evidence, as recorded in their minutes and register; and that it will be their especial duty to assist in rendering this important Act more practically tically applicable to circumstances in its execution, and still more conducive than it is at present to the public good.

I have the honour to be, my Lord,

Your Lordship's obedient Servant,

R. Powell, M.D. Sec.'

To the Right Hon. the Earl of Radnor,
&c. &c. &c."

"To the Right Honourable George Rose.

Sir, April 22, 1813.

I have the honour of communicating to you the papers to which I referred, respecting the Madhouse Act, and the following short account of the proceedings of the Commissioners upon that subject, with which it seemed proper that you should be acquainted. In the spring of the year 1811, Mr. William Wynne's bill for regulating Pauper and Criminal Lunatics, was brought before Parliament, for the purpose of introducing some amendments therein; and on that occasion Sir Lucas Pepys and I had some conversation with him upon the subject of the Act for regulating Madhouses, in consequence of which the Commissioners entered into a full consideration of the provisions of the Act, and made therein such alterations as they conceived likely to be practically beneficial: these were submitted to the opinion of Mr. Warren, the College counsel, and Lord Radnor has the only copy of his reply to the more general suggestions which were contained in the amended Act. The Act, as at that time altered, forms the chief part of the copy which Lord Radnor has now laid before you. The Commissioners then thought it to be their duty to call the attention of the College to the subject, and to require their determination upon the propriety of making an application to the legislature for the amendment of the Act; but, after the annual change of the College officers and Commissioners, the consideration passed by at the time; and it has now been renewed in consequence of certain circumstances which have occurred in Wiltshire, under the immediate notice of the Earl of Radnor, and have induced him to interest himself in the endeavour to prevent a repetition of them. Reports from the visitors in counties are very irregularly made; and one dated November 23, 1812, which refers to the subject before us, is the first which for many years back has been received from the county of Wilts. This Report states, that 23 persons were confined in a licensed house then visited, eight of whom were paupers sent by parish officers, and details many circumstances in its management, which deserved severe censure. On examining the register of lunatics admitted into such houses, it appeared that not one of the 15 patients who were not paupers had been returned to the secretary in London, for the insertion of the name therein, according to the directions of the Act; and this information was immediately stated officially from the Commissioners to the Clerk of the Peace for the county, but he has not noticed to them them the receipt of such communication. On March 13th last, Sir Francis Milman laid before the Commissioners a letter from Lord Radnor to Doctor Pemberton, enclosing a plan intended to obviate, in future, the evil which had been recently found to exist in Wiltshire; and at the same time requesting to know if the Commissioners had any suggestions to offer upon the same subject. The Commissioners thus applied to referred to the paper which had been drawn up in 1811, and made some additions thereto, in consequence of some circumstances of still later occurrence; but they judged, that, in answering Lord Radnor's letter, it would be desirable, in the first instance, to address a more general report to his Lordship, and to lay before him a view of the practical objections to the existing Act, and of those alterations which appeared to them to be necessary. A copy of this letter I have now the honour to transmit to you. Lord Radnor took the trouble to digest these suggestions into the form of the clauses of an Act of Parliament, which he returned for the consideration of the Commissioners, who, in order that their bearings and relations to the present Act might be more clearly seen, directed that such of them as had not been noticed on the former occasion, should be added thereto, and the whole entered in their proper places in an interleaved copy of the present Act. This copy was then sent to Lord Radnor, for his opinion and observations, and has been communicated by him to you. The Commissioners, however, are aware, from experience, that much more caution and attention will be necessary in wording the Act, so as to make it clear and definite in its practical application; and believe, that it would be better to form a new Act altogether, than to attempt to amend the old one; and they purposed, in the next place, to apply for the direction therein of some person especially conversant in the form and language of Acts of Parliament, and they submit it to you, that some such step will still be proper, before any application be made to the legislature.

I have the honour to be, Sir,

Your most obedient humble Servant,

R. Powell, M.D. Sec."

The importance of the above document cannot be questioned, nor is it for us to enquire into the powers given to the Commissioners; yet, under the most imperfect information, we cannot help expressing some surprise that so powerful a body as the London College should not refuse, *suo periculo*, licenses whenever they thought proper, and trust to the issue of any action from the parties who might think themselves aggrieved.

